# Regional differences in the utilisation of antenatal care and skilled birth attendant services during the COVID-19 pandemic in Nigeria: an interrupted time series analysis

**DOI:** 10.1136/bmjgh-2023-012464

**Published:** 2023-10-21

**Authors:** Rotimi Felix Afolabi, Mobolaji M Salawu, Eniola A Bamgboye, Segun Bello, Ayo Stephen Adebowale, Magbagbeola David Dairo, Steven N Kabwama, Irene Wanyana, Susan Kizito, Rawlance Ndejjo, Rhoda K Wanyenze, Olufunmilayo Ibitola Fawole

**Affiliations:** 1 Department of Epidemiology and Medical Statistics, Faculty of Public Health, College of Medicine, University of Ibadan, Ibadan, Nigeria; 2 Population and Health Research Entity, Faculty of Humanity, North-West University, Mafikeng, South Africa; 3 Department of Community Health and Behavioural Sciences, School of Public Health, College of Health Sciences, Makerere University, Kampala, Uganda; 4 Department of Epidemiology and Biostatistics, School of Public Health, College of Health Sciences, Makerere University, Kampala, Uganda; 5 Department of Disease Control and Environmental Health, School of Public Health, College of Health Sciences, Makerere University, Kampala, Uganda

**Keywords:** COVID-19, Public Health

## Abstract

**Introduction:**

The COVID-19 pandemic has had a substantial negative impact on the utilisation of essential health services (EHS) globally, especially in resource-limited settings such as Nigeria. High maternal deaths associated with low access to and utilisation of EHS such as antenatal care (ANC) and skilled birth attendants (SBAs) remain a concern during the COVID-19 era. The study assessed the COVID-19 pandemic effects on ANC and SBA utilisation across regions in Nigeria.

**Methods:**

Monthly data on ANC and SBA between January 2017 and July 2021 were obtained from the Federal Ministry of Health database. An interrupted time-series analysis, implemented using the Prophet model, was conducted to compare the regional variation of outcomes during the COVID-19 pandemic. Average percentage changes (PC) between the observed and predicted outcomes including their 95% CI were reported.

**Results:**

From March 2020 to July 2021, the number of ANC visits was significantly lower than expected by a 16%–43% change in five of the six regions in Nigeria. The highest significant reduction was in North-West (PC=−43.4; 95% CI: −52.6 to –34.1) and the least in South-West (PC=−15.5; 95% CI: −24.8 to –6.1), with no significant change in the South-East. The number of deliveries by SBA was significantly lower than expected by a 18%–43% change in all the regions (p<0.01). North-East (PC=−43.3; 95% CI: –51.7 to –34.9) and South-West (PC=−18.3; 95% CI: −25.2 to –11.5), respectively, had the highest and the least decline in SBA utilisation. Overall, ANC and SBA patterns of change were relatively similar across the north-south divide though the change effect was considerably pronounced in the north.

**Conclusion:**

There was a substantial reduction in ANC and SBA utilisation due to the COVID-19 pandemic in Nigeria, especially in the northern regions. Targeted and contextually relevant interventions should be implemented to alleviate the impact of emergency response on access to EHS and promote access to care during the pandemic.

**WHAT IS ALREADY KNOWN ON THIS TOPIC**

- The COVID-19 non-pharmaceutical interventions such as movement restrictions have been shown to disrupt routine essential health services (EHSs).
- Policy-makers use research evidence to make informed decisions on maintaining EHS during public health emergencies. Such evidence has included the impact of pandemics on the utilisation of EHS such as antenatal care (ANC).

**WHAT THIS STUDY ADDS**

- The COVID-19 pandemic substantially lowered ANC attendance and utilisation of skilled birth attendant (SBA) services in the six regions of Nigeria, although with some regional differences.
- The northern regions witnessed a significant higher decline in both ANC and SBA utilisation relative to the southern regions.
- The South-South region experienced the largest reduction in ANC utilisation, while the South-East region had the largest reduction in SBA at delivery among the southern regions.

**HOW THIS STUDY MIGHT AFFECT RESEARCH, PRACTICE OR POLICY**

- Implementing restrictions and precautions during public health emergencies in Nigeria requires careful consideration of the attendant impact on routine EHSs.
- The evidence emphasises the need to strengthen region-specific policies in light of sociocultural contexts that promote sustained access to quality maternal and new-born care during emergencies such as COVID-19 in Nigeria and other similar settings.

## Introduction

Public health emergencies including the COVID-19 pandemic often disrupt the delivery of essential health services (EHSs) including antenatal care (ANC) visits and facility deliveries by skilled birth attendants (SBA).^1 2^ Evidence suggests that some interventions in response to the COVID-19 pandemic especially the non-pharmaceutical interventions have considerably disrupted the provision of EHS, affecting the lives of millions of women and families.^3^ According to Riley *et al*,^4^ about a 10% reduction in pregnancy-related and neonatal healthcare coverage could increase maternal and neonatal mortalities, respectively, by 28 000 and 168 000 deaths worldwide. The disruptions have been attributed to distorted health workforce and supply chains, and decreased care-seeking behaviour for non-COVID-19 healthcare-related visits, among other factors.^5–7^ Besides, patients’ fear of acquiring an infection while in public places during the pandemic, especially in healthcare facilities reduces the demand for EHS and keeps people away from receiving healthcare.^8^ Worrisome, about a decade of progress in reproductive, maternal and child health could be stalled if not reversed.^6 9^

The COVID-19 pandemic has impacted on the safety of childbirth, access to treatment for complications in health facilities, and delivery of already-fragile maternal and child health services.^10^ The disruptions affected ANC, a major determinant of maternal mortality rate, and important components of maternal care^11–13^ including access to institutional-based deliveries.^2 4 6 10 14 15^ Proper utilisation of ANC considerably enhances safe motherhood with improved maternal outcomes. Skilled assistance at birth has been promoted over the years^14 16^ and has positively impacted on maternal health outcomes. However, ANC service utilisation may not necessarily suggest child delivery at an institutional-based facility.^3^ This implies that not only improved use of ANC services but also the presence of skilled birth assistants (SBA) at delivery should be targeted in the ongoing COVID-19 pandemic response, particularly in resource-limited settings such as Nigeria.

In 2018, a national survey in Nigeria reported that 67% of women who gave birth in the 5 years preceding the survey received ANC from a SBA at least once for their last birth.^17^ The onset of COVID-19 in Nigeria diverted human and financial resources from routine healthcare services to pandemic-response activities, particularly in the early part of 2020.^15 18^ Service utilisation was especially affected by the COVID-19 non-pharmaceutical interventions such as physical distance and movement restrictions (eg, curfews, partial and total lockdowns) and the fear of being infected with the virus at these health centres.^7^ Using data obtained from the health information system, studies have reported mixed results for the effect of COVID-19 pandemic-related disruptions on EHS.^19–21^ For instance, Arsenault *et al* 20 reported a range of 5%–33% significant decline in maternal health services including ANC visits and facility-based delivery in nearly half of the 10 countries studied. Similarly, a study among 12 sub-Saharan Africa countries observed about a 2%–6% modest reduction in the utilisation of maternal and child health services.^21^ These suggest varying impact of COVID-19 pandemic on EHS across countries and regions.

In Nigeria, researchers have reported a 26% reduction in health services access, a 16% drop in the number of ANC visits, and a 6% decrease in facility deliveries in the first half of 2020 relative to 2019 due to the COVID-19 pandemic.^15 22^ These disruptions could leave 700 000 women without access to facility-based delivery and consequently may result in childhood and maternal mortality rate increasing by 20% and 10%, respectively, over 1 year.^15^ Therefore, reducing preventable maternal and child deaths requires sustained access to quality maternal and newborn care. These previous studies were based on mathematical simulations or restricted to local or qualitative studies.^15 22^ This study analysed real-world national data which would provide more robust findings. Specifically, the study assessed the effect of the COVID-19 pandemic on ANC and SBA utilisation across the six regions in Nigeria using an interrupted time series (ITS) analysis. The findings of the study may highlight the importance of context-specific planning for robust maternity services in any public health emergency response.

## Methods

### Study design and setting

**Figure 1:**
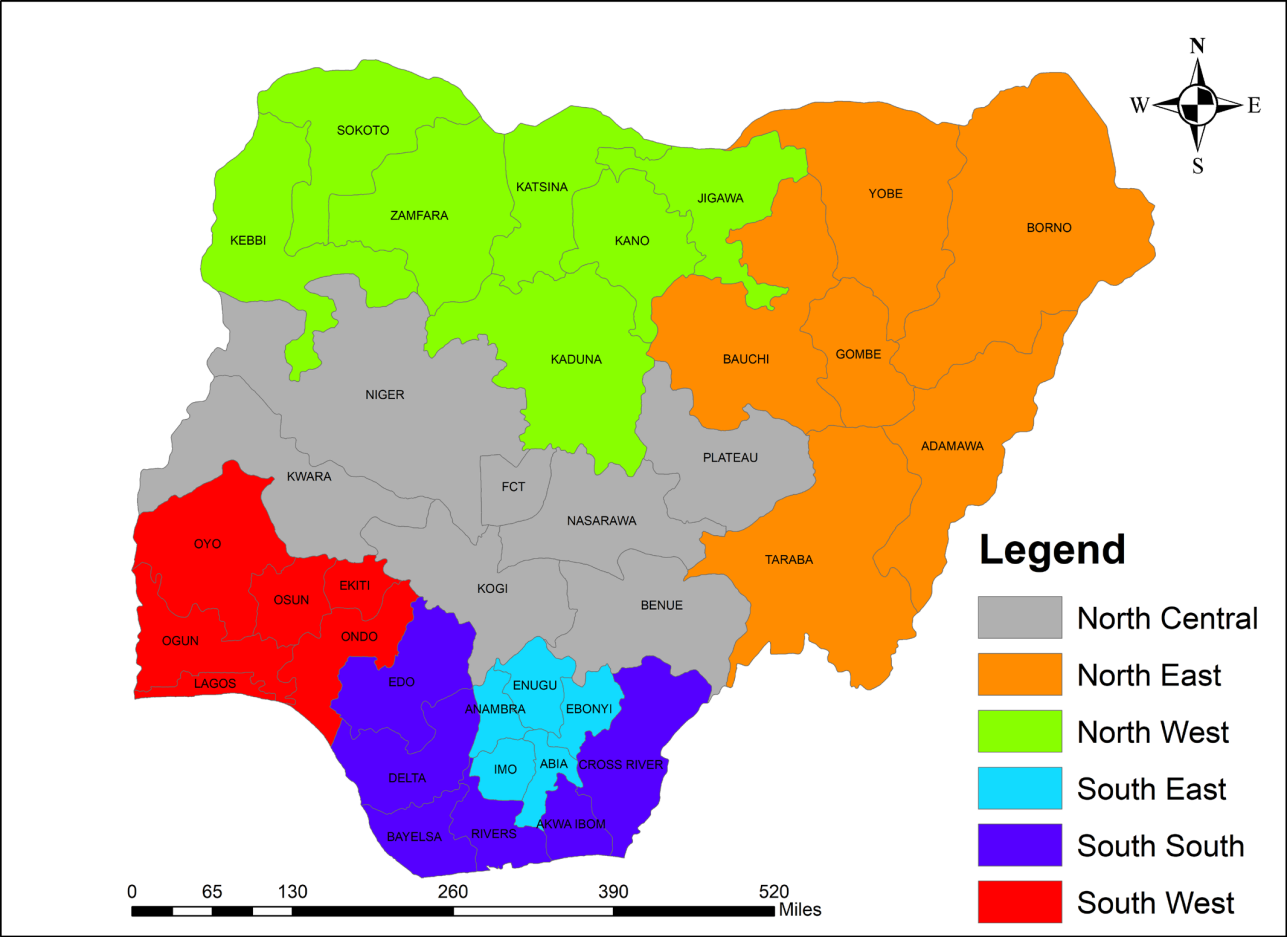
Map of Nigeria showing boundaries of six geopolitical zones.

This cross-sectional study was conducted to assess the effect of the COVID-19 pandemic on some EHS indicators in Nigeria. Nigeria is Africa’s most populous country and has a large, socioculturally diverse population of about 216 million.^23^ Administratively, Nigeria has 36 states including a Federal Capital Territory zoned into six geopolitical groups: namely, North-Central, North-East, North-West, South-East, South-South and South-West as shown in figure 1. Northern regions (North-Central, North-East and North-West) predominantly house Hausa/Fulani ethnic group and practise Islam religion; southern regions (South-East, South-South and South-West) are dominated by Christians of Igbo ethnic group in the South-East and Yoruba in the South-West.

Healthcare is being delivered at three levels which are tertiary, secondary and primary healthcare, respectively, managed by the federal, state and local governments. Nigeria has a low health workers-to-population ratio of 1.6 health workers including doctors, nurses and midwives per 1000 persons, below the WHO recommendation of 4.5 health workers for every 1000 persons.^22^ By 5 June 2022, Nigeria had recorded 256,250 COVID-19 cases and 3143 deaths with a 1.2% case fatality rate.^24^

### Data source and extraction

The secondary data used for the study were obtained from the health management information system—an electronic database of the Federal Ministry of Health (FMoH), Nigeria. The data were retrospectively extracted by FMoH staff who had access to the electronic medical records (EMR) system which operates on the District Health Information Software 2 (DHIS2). The nationally representative dataset provides information on several DHIS2 indicators including EHS. Specifically, the state-level data contained monthly information regarding the number of ANC visits and the number of deliveries by SBA in Nigeria from January 2017 to July 2021.

### Study variables

In this study, the exposure was the COVID-19 pandemic. Two of the key components of EHS were considered as outcome measures. These are the monthly number of ANC visits and deliveries by SBA across the six regions in Nigeria. Although the first COVID-19 case was confirmed in Nigeria on 27 February 2020, 1 March 2020 was used to define the outcome measures before and after the emergence of the disease—assuming a negligible change would have happened.

### Data management and analysis

#### Data preparation

The obtained state-level data consisted of a few missing data (1.9%) on ANC utilisation in the months of September–December 2020, across the regions but South-South. Of the 39 missing data, December (41.0%) has the highest, followed by November (38.5%) and the least (10.3%) both in September and October. To make the data conform with ITS analysis and address the identified challenges including under-reporting and over-reporting, the missing data were imputed with the mean of non-missing values. Further, a Hampel (*x, window_size=5, n=3, imputation=True*) function in Python was applied to detect outlier (defined as any observed value within a rolling window of size five greater than three median absolute deviations) and replace such with its rolling window’s median. Only a few outliers in the state-level data on ANC utilisation (3.8%) and on deliveries by SBA (2.1%) was flagged and replaced with the rolling median (see online supplemental appendix 1). The data were then aggregated into six regions according to the administrative geopolitical zones in Nigeria. This was plotted using time series plots to visualise regional differences in trend and any unusual features of the data.

10.1136/bmjgh-2023-012464.supp3 Supplementary data

Thereafter, the ITS analysis using Prophet model was employed to evaluate the potential impact of the COVID-19 pandemic related-disruption on the two EHS indicators with the assumption that the level and trend of each outcome would remain the same if there was no interruption (COVID-19 pandemic).

#### Interrupted time series

An ITS analysis is often used in public health to evaluate the impact of an interruption like an intervention or an exposure.^25 26^ In this study, the emergence of COVID-19 disease was regarded as an interruption. The ITS regression model was used to compare the regional variation of the predicted outcomes to the observed outcomes during the COVID-19 era. Modelling of the data in the pre-COVID-19 enables assessment of the underlying trend and yields a counterfactual for what could have happened in the absence of the COVID-19 pandemic when extrapolated into the post-COVID-19 onset. Provided the underlying trend has been accounted for, differences between counterfactual (predicted) and observed data at post-COVID-19 onset could be evaluated. The ITS analysis implemented using Facebook Prophet was employed. The data analysis was implemented using Python V.3.10 programming language and the level of significance was set at 5%.

#### The Prophet model approach

The Facebook’s Core Data Science team developed Prophet as a forecasting model.^27^ Prophet is robust to outliers and shifts in the trend and it can handle various seasons of historical time series data. Relative to other approaches, studies have recommended Prophet due to its open source algorithm, automated nature, accuracy, efficient and speedy time series analysis and forecasting.^28–30^ The forecasting model is an additive model where nonlinear trends are modelled with yearly, monthly, weekly or daily seasonality including holiday effects.

The Prophet model is described in equation (1)

(1) $$X(t)=T(t)+S(t)+H(t)+e_{t}$$

where:

X(t) is the outcome measure.

T(t) is the trend function (models non-periodic changes).

S(t) represents seasonality (models periodic changes).

H(t) represents effects of holidays and events (models often non-periodic shocks; this component was not considered in this study as the data do not have an effect of the holiday term in trend forecasts).

$e_{t}$ is an error term (models idiosyncratic changes).

Of note, Prophet employs a Fourier series to estimate the seasonality effects, and the seasonality models are specified as the periodic functions of time *t*.

#### Model hyperparameter

Hyperparameter tuning is the process of obtaining the best hyperparameters to improve model performance. The process is crucial for the optimal data training process; thus, the best set of hyperparameters is obtained before data training. After the data were preprocessed, a preliminary forecast was done to tune hyperparameters using a grid search cross-validation approach. The approach used a 4×4×2 grid of three parameters (changepoint prior scale (0.001, 0.01, 0.05, 0.1); seasonality prior scale (0.01, 0.1, 1.0, 10.0); seasonality mode (additive, multiplicative) to choose the best set of hyperparameters. The changepoint prior scale defines the trend flexibility, the seasonality prior scale defines the seasonality flexibility, while the seasonality mode identifies seasonality as either additive or multiplicative. With prediction date ranging from February 2019 to October 2019 and parallelisation over different periods, the hyperparameters were evaluated on root mean square error averaged over a 3-month horizon. The best combination of tuned parameters with the minimum RSME for each of the outcomes by region is presented in table 1.

**Table 1:** The tuned best-value parameters

Region	ANC visits			Deliveries by SBA		
	Changepoint prior scale	Seasonality prior scale	Seasonality mode	Changepoint prior scale	Seasonality prior scale	Seasonality mode
North-Central	0.05	0.01	additive	0.1	1.0	additive
North-East	0.1	0.01	additive	0.1	10.0	additive
North-West	0.001	1.0	additive	0.01	0.1	additive
South-East	0.01	0.01	additive	0.001	1.0	additive
South-South	0.001	0.1	multiplicative	0.05	10.0	multiplicative
South-West	0.1	1.0	multiplicative	0.001	10.0	additive

ANC, antenatal care; SBA, skilled birth attendant.

#### Modelling and forecasting

In practice, a model is often trained on observed data and used to predict the counterfactual.^29 31^ Therefore, the data were divided into two parts: training and testing sets. The training set was used to optimise and train a model such that the structured underlying pattern of the ordered data is obtained. The testing set was used to fit and evaluate a prediction model. Specifically, data up to February 2020 were used for training and validating the model using the best-tuned hyperparameters; thereafter, the model was applied to predict the outcome for the remaining months in the data (March 2020–July 2021). Predicted and observed data were plotted together to visualise the fitting accuracy of the model and quantified to assess the level of change due to COVID-19. The change was assessed using percentage change (PC), as expressed in equation (2)

(2) $$\;PC=\frac{1}{n}\sum_{t=1}^{n}\left(\frac{X_{t}-\hat{X_{t}}}{\hat{X_{t}}}\right)\times \%$$

where: $X_{t}$ = actual value at time t; ${\hat{X}}_{t}$ = predicted value at time t; n=number of data points

Additionally, the test of significance for the differences between the observed and predicted outcome’s mean counts was conducted using the Wilcoxon signed-rank test. The 95%CIs for PC including the corresponding p values were reported.

#### Model prediction accuracy

The prediction accuracy of a model using a cross-validation approach was conducted. Such that predictions were made for each of the outcomes during COVID-19 pandemic on a 3-month horizon. The prediction performance was evaluated by comparing the trends in the actual and predicted outcomes using the mean absolute percentage error (MAPE). This was calculated on a rolling window of the predictions after sorting by horizon. The MAPE is a commonly used performance metric to evaluate modelling capability and predictive ability. It is a measure of percentage errors in prediction, expressed as in equation (3).

(3) $$MAPE=\frac{1}{n}\sum_{t=1}^{n}\left|\frac{X_{t}-\hat{X_{t}}}{X_{t}}\right|\times \%$$

The MAPE is an easy-to-interpret scale-independent measure that compares prediction performance between various scaled datasets.^28 31 32^ A forecast’s MAPE value ≤10% is interpreted as highly accurate, 11%–20% good, 21%–50% reasonable and >50% inaccurate forecasting.^33^

### Patient and public involvement statement

Patients or the public were not involved in the design, implementation, reporting or dissemination plans of this study

## Results

### The trend of ANC and SBA utilisation measures by region between January 2017 and July 2021 in Nigeria

**Figure 2:**
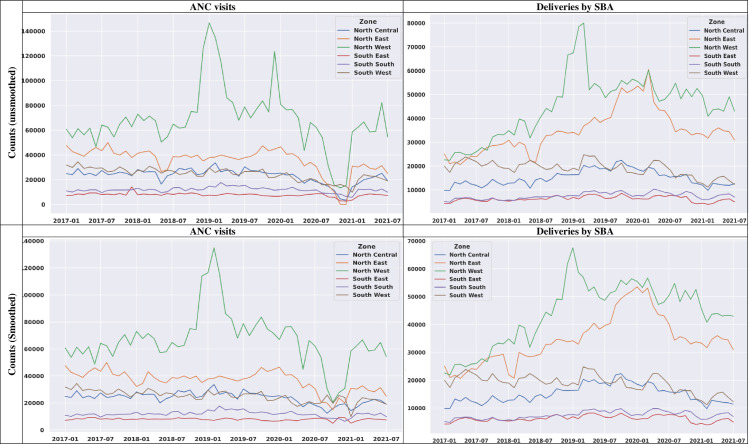
Regional trend for monthly observed counts of ANC visits and deliveries by SBA. ANC, antenatal care; SBA, skilled birth attendant.

The trends of the observed monthly counts of ANC visits and deliveries by SBA are presented in figure 2. The highest number of ANC visits was observed in North-West, followed by North-East, while the least count was observed in South-East though the trend was similar to that of South-South. Except for 2017 when South-West had relatively lower counts compared with North-Central, the two regions had relatively the same trend of ANC utilisation. From January 2020, a decrease in the count was observed in most regions, prominently in the North-West and North-East regions. The observed decline in counts was, however, reversed starting from October 2020.

An increasing trend in the number of deliveries by SBA was observed prominently in the northern regions. This, however, was reversed at the onset of the pandemic with a decrease in the monthly counts for SBA utilisation at delivery. Similar to the trend in ANC utilisation, the North-West had the highest number of deliveries by SBA, followed by the North-East and South-West regions.

### Visualisation of the fitting accuracy of the model across the regions in Nigeria

#### Predicted and observed counts of ANC visits comparison

**Figure 3:**
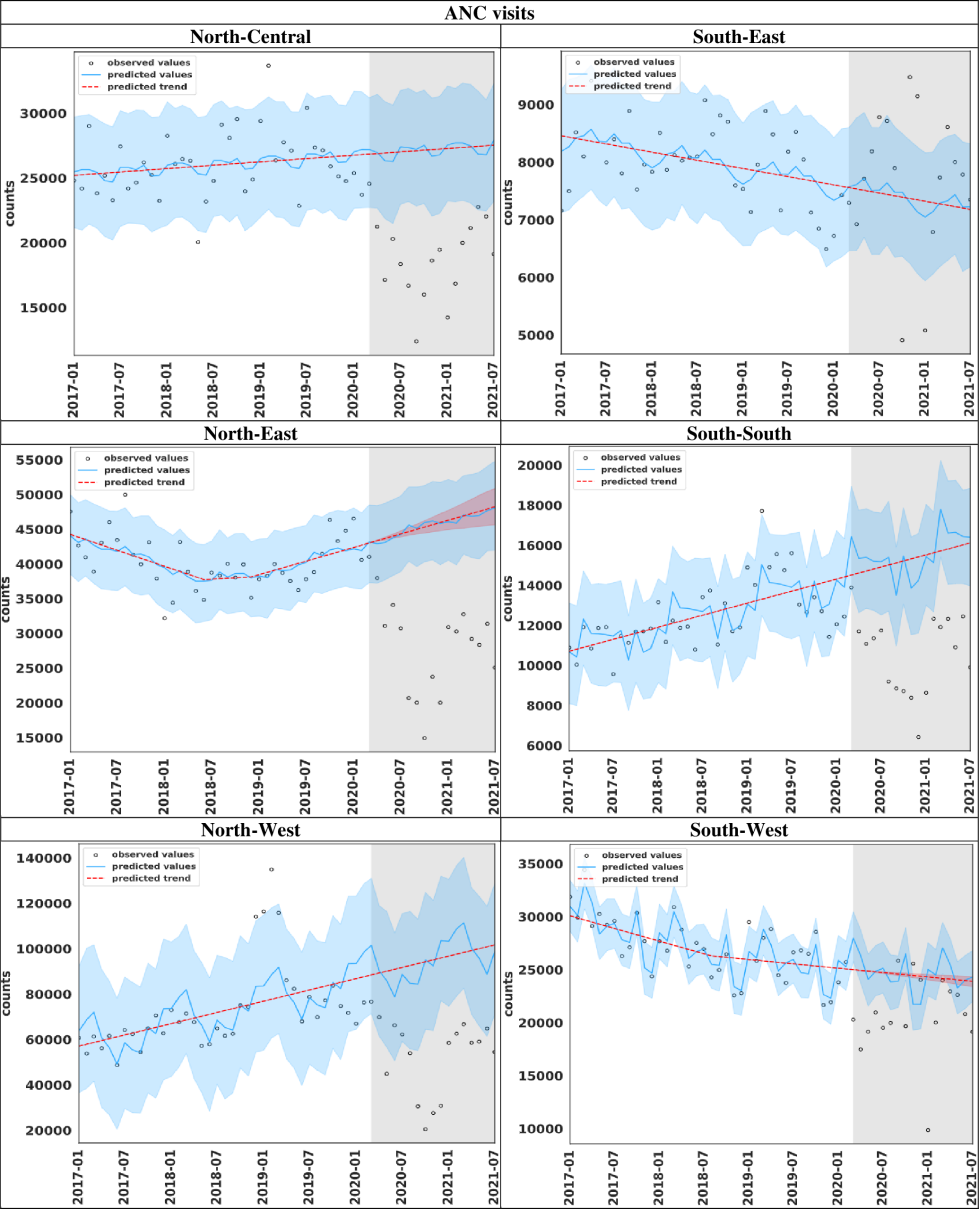
ITS plots showing the observed and predicted monthly values of ANC visits according to regions in Nigeria. Note: The blue portion indicates the 95% CI for prediction, while the grey area indicates the COVID-19 era (March 2020–July 2021). ANC, antenatal care; ITS, interrupted time series.

The fitted accuracy of the model for ANC utilisation using both the observed and cumulative counts are shown in figure 3 and Online supplemental figure 1, respectively. The models, for each of the regions, perform well as most observed counts are within the 95% CI for the predicted values before the emergence of the COVID-19 pandemic. If there were no COVID-19 outbreak, the monthly reported number of ANC visits was expected to increase in all the regions, except for South-East and South-West regions that had downward trend movements. Additionally, the observed monthly ANC visits nosedived during COVID-19 in most regions, but South-East region (figure 3). Corroborating the fitting accuracy of the model, the predicted and observed cumulative trend values were approximately the same pre-COVID-19 emergence period in all the regions (see online supplemental figure 1). Reduced ANC utilisation was observed post-COVID-19 emergence in all regions, except the South-East region, as the expected monthly cumulative number of ANC visits was respectively higher than the observed values.

10.1136/bmjgh-2023-012464.supp1 Supplementary data

#### Predicted and observed counts of deliveries by SBA comparison

**Figure 4:**
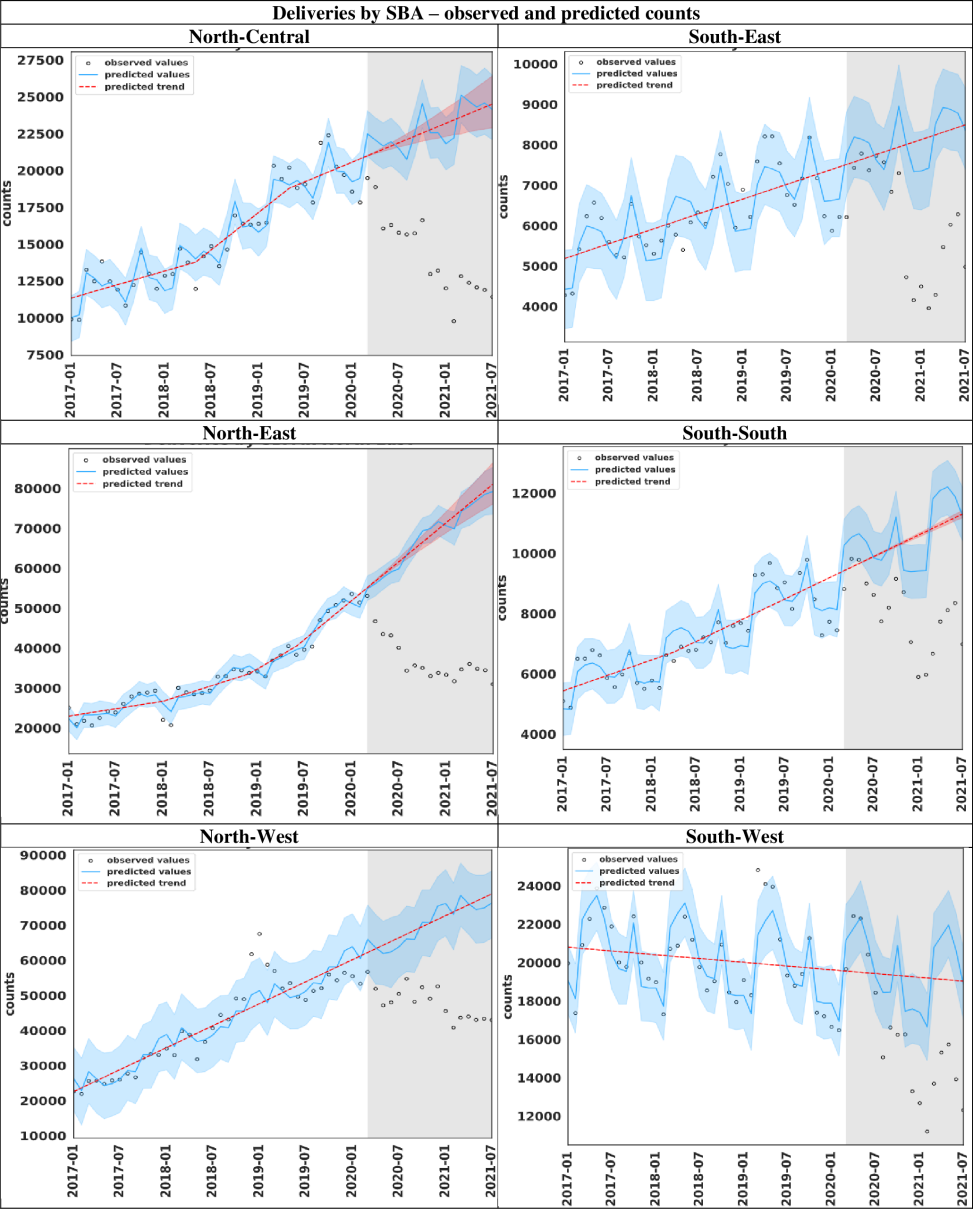
ITS plots showing the observed and predicted monthly values of deliveries by SBA according to regions in Nigeria. Note: The blue portion indicates the 95% CI for prediction, while the grey area indicates the COVID-19 era (March 2020–July 2021). ITS, interrupted time series; SBA, skilled birth attendant.

The fitted accuracy of the models for SBA utilisation at delivery, comparing both the observed and cumulative counts with predicted values in each of the regions are respectively shown in figure 4 and online supplemental figure 2. Before the emergence of the COVID-19 pandemic, the models fit well as most observed counts for deliveries by SBA are within the 95% CI for the prediction (figure 4). If there was no COVID-19 outbreak, the monthly reported number of deliveries by SBA was expected to increase in all regions except South-West region. The observed monthly deliveries by SBA decreased sharply at the onset and during the COVID-19 pandemic in the northern regions relatively compared with the southern regions (figure 4). Similarly, predicted and observed cumulative trend values were approximately the same pre-COVID-19 emergence in all the regions (see online supplemental figure 2). The counterfactual monthly cumulative number of deliveries by SBA was respectively higher than the observed values in all regions.

10.1136/bmjgh-2023-012464.supp2 Supplementary data

### Assessment of the level of change in outcome measures due to COVID-19

**Figure 5:**
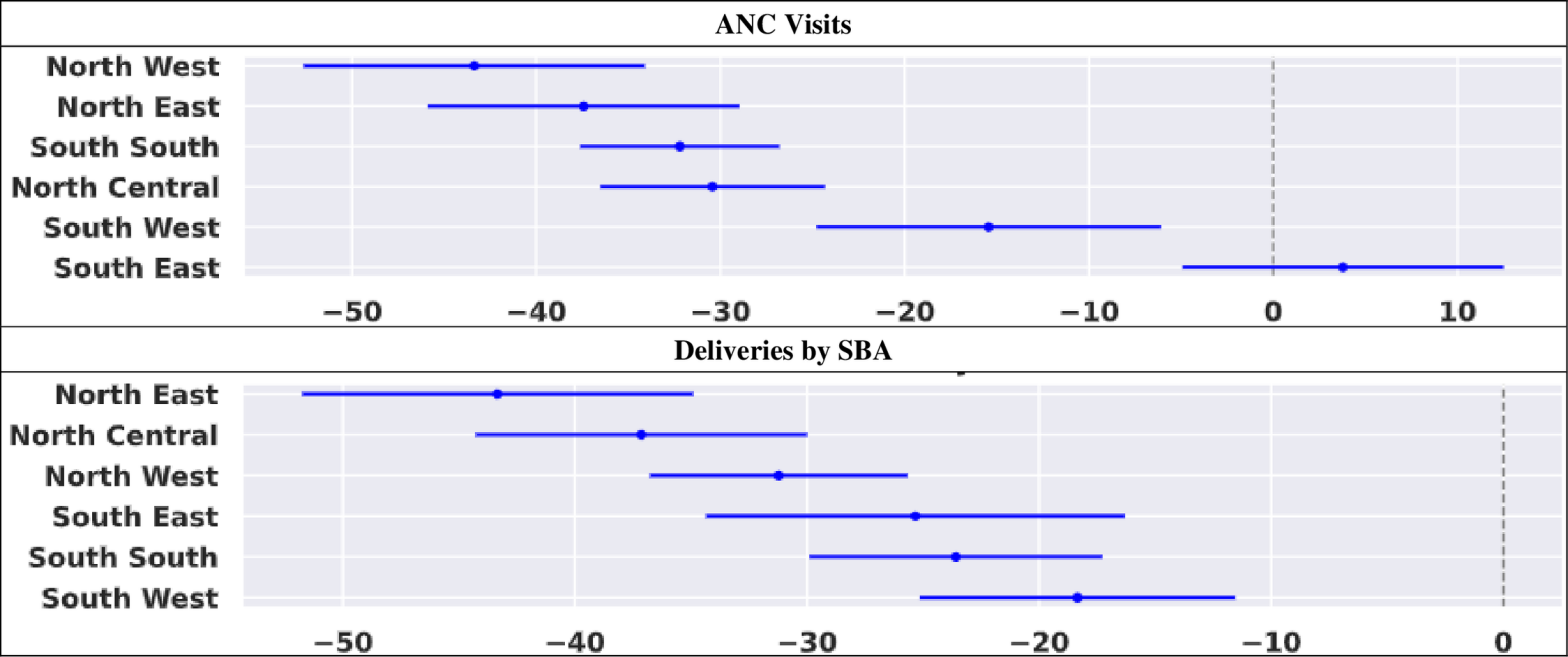
Percentage change in predicted and observed mean counts for ANC visits and deliveries by SBA according to regions in Nigeria (March 2020–July 2021). ANC, antenatal care; SBA, skilled birth attendant.

The PC in average counts of ANC visits and deliveries by SBA, within March 2020 and July 2021, respectively, are shown in figure 5. The chart reveals negative PC between the observed and the predicted mean counts for both outcomes in most regions. Table 2 also corroborates these findings.

**Table 2:** Differences between the observed and predicted outcome’s mean counts by regions in Nigeria

Region	ANC visits’ average counts				Deliveries by SBA average counts			
	Actual	Predicted	PC (CI)	P value*	Actual	Predicted	PC (CI)	P value*
North-Central	18 881	27 167	30.4 (–36.5 to 24.4)	<0.001	14 300	22 912	37.1 (–44.3 to 30.0)	<0.001
North-East	28 375	45 567	37.4 (–45.9 to 29.0)	<0.001	37 313	67 858	43.3 (–51.7 to 34.9)	<0.001
North-West	53 425	94 853	43.4 (–52.6 to 34.1)	<0.001	47 915	70 392	31.2 (–36.8 to 25.7)	<0.001
South-East	7671	7392	3.8 (–4.9 to 12.5)	<0.190	6036	8100	25.3 (–34.4 to 16.3)	<0.001
South-South	10 580	15 560	32.2 (–37.6 to 26.8)	<0.001	8036	10 575	23.6(–29.9 to 17.3)	<0.001
South-West	20 701	24 692	15.5 (–24.8 to 6.1)	0.002	16 208	19 774	18.3 (–25.2, to 11.5)	<0.001

CI: 95% CI for PC.

*P value based on Wilcoxon sign-ranked test.

ANC, antenatal care; PC, percentage change; SBA, skilled birth attendant.

#### ANC utilisation

The PCs in the number of ANC visits between observed and predicted values were significant in all regions but in South-East. The significant negative PC (p<0.001) in ANC utilisation observed in South-South (PC=−32.2; 95% CI: –37.6 to –26.8) was similar to that of the northern regions (North-East—PC=−37.4, 95% CI: –45.9 to –29.0; North-Central—PC=−30.4; 95% CI: –36.5 to –24.4; North-West—PC=−43.4; 95% CI: –52.6 to –34.1) as their 95% CI overlapped in figure 5. The change effect was, however, different from that of other southern regions.

#### SBA utilisation

In all the regions, a significant negative PC (p<0.001) in the mean difference of deliveries by SBA was observed with the highest decline in North-East (PC=−43.3; 95% CI: –51.7 to –34.9) and the least in South-West (PC=−18.3; 95% CI –25.2 to –11.5). Figure 5 reveals that the pattern of changes in observed and predicted mean counts for SBA utilisation was similar among the northern regions and the southern regions as their 95% CI overlapped, respectively.

### Evaluation of model performance

The model performance metric—MAPE is presented in table 3. A high-accurate prediction of ANC utilisation was observed in all regions (MAPE<10%) except for the North-West (MAPE=15.5%) and South-South (MAPE=11.2%) regions with a good prediction for about 3 months. The prediction accuracy improved from a month to 3-month horizon forecast for ANC utilisation nearly in all the regions. The North-Central (MAPE=1.1%), South-East (MAPE=2.6%) and South-West (MAPE=1.4%), respectively, had a low error rate indicating a very good prediction (table 3).

**Table 3:** Model performance metric based on MAPE

Region	MAPE					
	ANC visits			SBA at delivery		
	1 month	2 months	3 months	1 month	2 months	3 months
North-Central	6.4	0.2	1.1	4.0	4.5	9.6
North-East	7.4	8.3	6.1	5.9	9.4	13.3
North-West	15.4	12.1	15.5	10.9	12.4	3.4
South-East	6.3	9.3	2.6	8.2	11.2	11.9
South-South	10.8	9.9	11.2	3.1	0.4	0.8
South-West	4.1	8.5	1.4	4.5	10.0	8.9

ANC, antenatal care; MAPE, mean absolute percentage error; SBA, skilled birth attendant.

A high-accurate prediction (MAPE<10%) of SBA utilisation at delivery for the quarterly predictions was observed in the South-West, South-South, North-Central and North-West regions, while a good prediction was observed in North-East (MAPE<13.3%) and South-East (MAPE<11.9%) regions. The error rate improved from being good to a high-accurate prediction for the quarterly prediction in North-West, while the reverse scenario was observed in North-East and South-East regions. A low error rate, indicating a very good prediction, was observed in both South-South (MAPE=0.8%) and North-West (MAPE=3.4%) in all the horizons (table 3).

## Discussion

This study assessed the influence of the COVID-19 pandemic on the utilisation of ANC and SBA services across the six regions in Nigeria between March 2020 and July 2021. The significant negative PC in the utilisation of the EHS indicators in this study suggests that the COVID-19 pandemic substantially lowered ANC attendance and utilisation of SBA services in the six regions of Nigeria. Regional differences in the effects of COVID-19 on the utilisation of ANC and SBA services were observed with a reduction of service utilisation in the northern regions of Nigeria compared closely with each other. Due to a long-standing sociocultural belief system in most parts of northern Nigeria, the demand for health services had always been lower and more elastic than the demand from southern Nigeria. A typical example is reflected in the vaccination coverage which had been perennially lowered in the North with a DPT3 coverage as low as less than 20% in some northern states.^34^ It is not surprising that any disruptions in routine health services would produce a higher and longer-lasting impact in most parts of Nigeria’s northern regions.

Of note, the northern region with a lower COVID-19 pandemic prevalence^35^ compared with the southern region has the highest volume of EHS indicators utilisation as shown in this study. The highest volume recorded in the northern regions may be associated with the higher population in the regions relative to the southern regions. The northern regions, however, experienced a relatively substantial decline in both ANC and SBA utilisation. This suggests that the extent of EHS disruptions was not directly linked to the COVID-19 disease burden. North-south divide in Nigeria’s population could indeed be linked with the observed regional differences in EHS utilisation due to the COVID-19 pandemic and its policy responses. This emphasises the need to strengthen region-specific policies that promote sustained access to quality maternal and newborn care during emergencies such as the COVID-19 pandemic in Nigeria.

The health-system shocks such as the COVID-19 pandemic and its response have been connected to a reduction in EHS demand and delivery. In this study, the numbers of ANC visits and deliveries by SBA were substantially lower between March 2020 and July 2021 than the expected figures in the absence of the COVID-19 pandemic in five of the six regions of Nigeria. Nigeria, such as many countries across the globe, has experienced COVID-19-related morbidity and mortality among healthcare workers, leading to EHS delivery staff shortages.^22^ This shortage could cause delay in EHS access and poor usage of approved health facilities, among others. Before the COVID-19 pandemic, Nigeria’s health system was weak in terms of EHS delivery. It is possible that the pandemic, and perhaps the lockdown and other non-pharmaceutical interventions implemented to mitigate the spread of the disease further undermined the access to and utilisation of the EHS in Nigeria.^36^ Other studies^7 11 37 38^ have alluded to several other reasons such as drug stock-out, harassment by law enforcement workers, and transportation difficulties as attributable to a significant reduction in the utilisation of EHS during the COVID-19 pandemic.

In this study, ANC services utilisation declined by 16%–43%, while SBA at deliveries reduced by 18%–43%. This finding was in agreement with a study among 12 countries in sub-Saharan Africa including Nigeria^21^ that reported a reduction in the utilisation of EHS. Even though the impact of the COVID-19 pandemic on EHS access was comparable in the northern regions, South-South was the only southern region with a substantial impact on its ANC visits. This may be due to the lower levels of ANC utilisation in these regions compared with South-East and South-West as previously reported in studies conducted in Nigeria.^39 40^ As a matter of fact, the northern region is noted for poor healthcare-seeking behaviour which got worsened during the pandemic. The observed differences may further be compounded during the pandemic due to the higher incidence of poverty in the northern regions where healthcare systems are more likely to be inadequate or fragile. The link between access to care and poverty remains. This may critically impact on EHS affordability, availability and accessibility thereof. However, in the later part of the period when movement restriction including lockdowns were lifted, failure to seek care predominated.

The impact of COVID-19 related disruption on the use of SBA at delivery was substantially higher in the northern regions relative to the southern regions. The simultaneous reduction in ANC and SBA utilisation in the northern regions may suggest a decrease in demand for EHS. The associated barriers to decreased care-seeking behaviours such as distorted health workforce and supply chains, fragile health facilities, poor access and fear of infection have been reported in the literature globally.^5–8^ The varying degree of regional differences in the reduction of ANC utilisation may be compounded by cultural and socioeconomic differences such as educational attainment, religion and ethnicity, especially along the north-south divide of Nigeria, as suggested in previous studies.^39 41 42^ Additionally, instability and conflict such as Boko Haram insurgence (predominantly in North-East), Banditry (North-West), herdsmen and farmer clash (North-Central), and militancy (South-South) may further explain the reduction in EHS utilisation.^43 44^

### Study limitations and strengths

A few limitations were observed in this study. First, the data from EMR could have provided an incomplete picture of the actual values of each outcome measure due to reporting bias and missing values. Second, the obtained data constrained the exploration of additional variables while assessing the impact of the pandemic on the EHS indicators. Of note, missing data issues are still a concern but gradually reducing. Even though health facility reports are known to have multiple data quality issues, evidence of improvements abounds.^21^ Third, the representativeness of the DHIS data is controversial, especially with limited clear-cut subnational denominators. Thus, it would be unclear whether the declines in service utilisation could be entirely ascribed to failure to seek care or whether there was a factual less need to seek care. Furthermore, the lack of subnational denominators restricted more granular analysis to clarify whether any increase or decrease in the number of pregnancies (and by extension, ANC service utilisation) by late 2020, was attributable to COVID-19. Nonetheless, the study has been strengthened using a large nationally representative dataset. Besides, the strength of the study includes the use of the Facebook Prophet model which is robust to outliers and shifts in the trend, and of high accuracy for prediction.^22–24^ These enhanced reliable inferences. Also, the quantification of the regional variations in the level of change due to the COVID-19 pandemic in the utilisation of the EHS indicators remains a strength of this study.

## Conclusions

Regional variation and substantial decline in the utilisation of ANC and SBA were observed in Nigeria during the COVID-19 pandemic in March 2020–July 2021. While the northern regions witnessed a significant decline in both ANC and SBA utilisation due to the COVID-19 pandemic, South-South experienced the most reduction in ANC utilisation and South-East the most reduction in SBA at delivery among the southern regions. These findings highlight the need for context-specific interventions including tracking of disruptions of EHS and appropriate mitigation during disease outbreaks and pandemics. The forecasting results have the potential to assist governments and stakeholders in policy decisions to maintain progress towards achieving the Sustainable Development Goal health targets by 2030.

## Data Availability

Data are available on reasonable request. Data analysed in this study were obtained from DHIS2, domiciled with the FMoH Nigeria. Readers interested in replicating the analysis may contact the FMoH Nigeria for access to the data.

## References

[1] World Health Organization . Maintaining essential health services: operational guidance for the COVID-19 context, interim guidance. 2020. Available: https://www.who.int/publications/i/item/WHO-2019-nCoV-essential_health_services-2020.2 [Accessed 14 Apr 2022].

[2] Kc A , Gurung R , Kinney MV , et al . Effect of the COVID-19 pandemic response on Intrapartum care, Stillbirth, and neonatal mortality outcomes in Nepal: a prospective observational study. Lancet Glob Health 2020;8:e1273–81. 10.1016/S2214-109X(20)30345-4 32791117 PMC7417164

[3] Homer CSE , Leisher SH , Aggarwal N , et al . Counting stillbirths and COVID 19—there has never been a more urgent time. Lancet Global Health 2021;9:e10–1. 10.1016/S2214-109X(20)30456-3 33212029 PMC10011432

[4] Riley T , Sully E , Ahmed Z , et al . Estimates of the potential impact of the COVID-19 pandemic on sexual and reproductive health in low- and middle-income countries. Int Perspect Sex Reprod Health 2020;46:73–6. 10.1363/46e9020 32343244

[5] Chmielewska B , Barratt I , Townsend R , et al . Effects of the COVID-19 pandemic on maternal and perinatal outcomes: a systematic review and meta-analysis. Lancet Global Health 2021;9:e759–72. 10.1016/S2214-109X(21)00079-6 33811827 PMC8012052

[6] Hedstrom A , Mubiri P , Nyonyintono J , et al . Impact of the early COVID-19 pandemic on outcomes in a rural ugandan neonatal unit: a retrospective cohort study. PLoS ONE 2021;16:e0260006. 10.1371/journal.pone.0260006 34914748 PMC8675646

[7] Adelekan B , Goldson E , Abubakar Z , et al . Effect of COVID-19 pandemic on provision of sexual and reproductive health services in primary health facilities in Nigeria: a cross-sectional study. Reprod Health 2021;18:166. 10.1186/s12978-021-01217-5 34348757 PMC8334336

[8] Kwaghe AV , Kwaghe VG , Habib ZG , et al . Stigmatization and psychological impact of COVID-19 pandemic on frontline healthcare workers in Nigeria: a qualitative study. BMC Psychiatry 2021;21:518. 10.1186/s12888-021-03540-4 34670530 PMC8528377

[9] United Nations . Goal 3 | Department of economic and social affairs. 2021. Available: https://sdgs.un.org/goals/goal3 [Accessed 10 Apr 2022].

[10] de Barra M , Gon G , Woodd S , et al . Understanding infection prevention behaviour in maternity wards: a mixed-methods analysis of hand hygiene in Zanzibar. Soc Sci Med 2021;272:113543. 10.1016/j.socscimed.2020.113543 33578309 PMC7938378

[11] Ogunkola IO , Adebisi YA , Imo UF , et al . Impact of COVID-19 pandemic on antenatal healthcare services in sub-Saharan Africa. Public Health Pract (Oxf) 2021;2:100076. 10.1016/j.puhip.2021.100076 34151307 PMC8204802

[12] Sudhinaraset M , Landrian A , Cotter SY , et al . Improving stigma and psychosocial outcomes among post-abortion Kenyan women attending private clinics: a randomized controlled trial of a person-centered mobile phone-based intervention. PLoS One 2022;17:e0270637. 10.1371/journal.pone.0270637 35749557 PMC9232159

[13] Kuhnt J , Vollmer S . Antenatal care services and its implications for vital and health outcomes of children: evidence from 193 surveys in 69 low-income and middle-income countries. BMJ Open 2017;7:e017122. 10.1136/bmjopen-2017-017122 PMC5695442 29146636

[14] Bassey U , Oyewande A , Chukwunonye A , et al . Trends in the use of skilled birth attendants among women of reproductive age in a resource-limited setting. MGM J Med Sci 2022;9:19. 10.4103/mgmj.mgmj_78_21

[15] Global Financing Facility . Preserve essential health services during the COVID-19 pandemic. Nigeria; 2020. Available: https://www.globalfinancingfacility.org/country-briefs-preserve-essential-health-services-during-covid-19-pandemic [Accessed 25 Apr 2022].

[16] Walker T , Woldegiorgis M , Bhowmik J . Utilisation of skilled birth attendant in low-and middle-income countries: trajectories and key sociodemographic factors. Int J Environ Res Public Health 2021;18:10722. 10.3390/ijerph182010722 34682468 PMC8535845

[17] National Population Commission (NPC), ICF International . Nigeria demographic and health survey 2018. Abuja, Nigeria, and Rockville, Maryland, USA; 2019. Available: https://www.dhsprogram.com/ [Accessed 05 Nov 2019].

[18] Tesema A , Joshi R , Abimbola S , et al . Addressing barriers to primary health-care services for noncommunicable diseases in the African region. Bull World Health Organ 2020;98:906–8. 10.2471/BLT.20.271239 33293751 PMC7716106

[19] Doubova SV , Leslie HH , Kruk ME , et al . Disruption in essential health services in Mexico during COVID-19: an interrupted time series analysis of health information system data. BMJ Glob Health 2021;6:e006204. 10.1136/bmjgh-2021-006204 34470746 PMC8413469

[20] Arsenault C , Gage A , Kim MK , et al . COVID-19 and resilience of healthcare systems in ten countries. Nat Med 2022;28:1314–24. 10.1038/s41591-022-01750-1 35288697 PMC9205770

[21] Nichols E , Pettrone K , Vickers B , et al . Mixed-methods analysis of select issues reported in the 2016 world health organization verbal autopsy questionnaire. PLoS One 2022;17:e0274304. 10.1371/journal.pone.0274304 36206230 PMC9543875

[22] Kabwam SN , Kiwanuka SN , Fawole OI , et al . Essential health services. Nigeria Ex Glob Heal; 2022. Available: https://www.exemplars.health/emerging-topics/epidemic-preparedness-and-response/essential-health-services/nigeria [Accessed 23 May 2022].

[23] United Nations Department of Economic and Social Affairs Population Division . World population prospects 2022: summary of results. New York; 2022.

[24] Nigeria Centre for Disease Control . An update of COVID-19 outbreak in Nigeria. 2022. Available: https://ncdc.gov.ng/diseases/sitreps/?cat=14&name=An%20update%20of%20COVID-19%20outbreak%20in%20Nigeria [Accessed 13 Jul 2022].

[25] Turner SL , Karahalios A , Forbes AB , et al . Comparison of six statistical methods for interrupted time series studies: empirical evaluation of 190 published series. BMC Med Res Methodol 2021;21:134. 10.1186/s12874-021-01306-w 34174809 PMC8235830

[26] Ogallo W , Wanyana I , Tadesse GA , et al . Quantifying the impact of COVID-19 on essential health services: a comparison of interrupted time series analysis using prophet and poisson regression models. J Am Med Inform Assoc 2023;30:634–42. 10.1093/jamia/ocac223 36534893 PMC10018265

[27] Taylor SJ , Letham B . Forecasting at scale. Am Stat 2018;72:37–45. 10.1080/00031305.2017.1380080

[28] Liço L , Enesi I , Jaiswal H . Predicting customer behavior using prophet algorithm in A real time series Dataset. Eur Sci J 2021;17:10–20. 10.19044/esj.2021.v17n25p10

[29] Kumar N , Susan S . COVID-19 pandemic prediction using time series forecasting models. 2020 11th Int Conf Comput Commun Netw Technol ICCCNT 2020; 2020

[30] Aditya Satrio CB , Darmawan W , Nadia BU , et al . Time series analysis and forecasting of Coronavirus disease in Indonesia using ARIMA model and PROPHET. Procedia Comput Sci 2021;179:524–32. 10.1016/j.procs.2021.01.036

[31] Omran NF , Abd-el Ghany SF , Saleh H , et al . Applying deep learning methods on time-series data for forecasting COVID-19 in Egypt, Kuwait, and Saudi Arabia. Complexity 2021;2021:1–13. 10.1155/2021/6686745

[32] Montaño Moreno JJ , Palmer Pol A , Sesé Abad A , et al . Using the R-MAPE index as a resistant measure of forecast accuracy. Psicothema 2013;25:500–6. 10.7334/psicothema2013.23 24124784

[33] Tariq H , Hanif MK , Sarwar MU , et al . Employing deep learning and time series analysis to tackle the accuracy and robustness of the forecasting problem. Secur Commun Netw 2021;2021:1–10. 10.1155/2021/5587511

[34] Adegboye OA , Alele FO , Pak A , et al . A resurgence and re-emergence of diphtheria in Nigeria, 2023. Therapeutic Advances in Infection 2023;10:204993612311619. 10.1177/20499361231161936 PMC10061628 37008790

[35] Moroh JE , Innocent DC , Chukwuocha UM , et al . Seasonal variation and geographical distribution of COVID-19 across Nigeria (March 2020–July 2021). Vaccines (Basel) 2023;11:298. 10.3390/vaccines11020298 36851175 PMC9967289

[36] Asefa A , Semaan A , Delvaux T , et al . The impact of COVID-19 on the provision of respectful maternity care: findings from a global survey of health workers. women and Birth. Women and Birth 2022;35:378–86. 10.1016/j.wombi.2021.09.003 34531166 PMC9179099

[37] Burt JF , Ouma J , Lubyayi L , et al . Indirect effects of COVID-19 on maternal, neonatal, child, sexual and reproductive health services in Kampala, Uganda. BMJ Glob Health 2021;6:e006102. 10.1136/bmjgh-2021-006102 PMC8406460 34452941

[38] Aranda Z , Binde T , Tashman K , et al . Disruptions in maternal health service use during the COVID-19 pandemic in 2020: experiences from 37 health facilities in low-income and middle-income countries. BMJ Glob Health 2022;7:e007247. 10.1136/bmjgh-2021-007247 PMC8753094 35012970

[39] Fagbamigbe AF , Idemudia ES . Barriers to antenatal care use in Nigeria: evidences from non-users and implications for maternal health programming. BMC Pregnancy Childbirth 2015;15. 10.1186/s12884-015-0527-y PMC4407543 25885481

[40] Adewuyi EO , Auta A , Khanal V , et al . Prevalence and factors associated with underutilization of antenatal care services in Nigeria: a comparative study of rural and urban residences based on the 2013 Nigeria demographic and health survey. PLoS One 2018;13:e0197324. 10.1371/journal.pone.0197324 29782511 PMC5962076

[41] Sinai I , Anyanti J , Khan M , et al . Demand for women’s health services in northern Nigeria: a review of the literature. Afr J Reprod Health 2017;21:96–108. 10.29063/ajrh2017/v21i2.11 29624944

[42] Solanke BL , Rahman SA . Multilevel analysis of factors associated with assistance during delivery in rural Nigeria: implications for reducing rural-urban inequity in skilled care at delivery. BMC Pregnancy Childbirth 2018;18. 10.1186/s12884-018-2074-9 PMC6225672 30409121

[43] Chukwuma A , Ekhator-Mobayode UE . Armed conflict and maternal health care utilization: evidence from the Boko Haram insurgency in Nigeria. Soc Sci Med 2019;226:104–12. 10.1016/j.socscimed.2019.02.055 30851661

[44] Solanke BL . Factors associated with use of maternal Healthcare services during the Boko Haram insurgency in North-East Nigeria. Med Confl Surviv 2018;34:158–84. 10.1080/13623699.2018.1511358 30156121

