# Supplementary material for: Regional differences in the utilisation of antenatal care and skilled birth attendant services during the COVID-19 pandemic in Nigeria: an interrupted time series analysis
Source: BMJ Glob Health. 2023 Oct 21;8(10):e012464. doi: 10.1136/bmjgh-2023-012464 (PMC10603444; doi:10.1136/bmjgh-2023-012464)
Supplement: Supplementary data [file bmjgh-2023-012464supp003.pdf]

Appendix 1 Distribution of the state-level data missingness and detected outliers by region according to pre- and during-COVID-19 era

Region	No of States	Pre-COVID-19 (January 2017 – February 2020)					During COVID-19 (March 2020 – July 2021)					Overall (January 2017 – July 2021)				
		Detected Outlier		Missing observation		No of observation	Detected Outlier		Missing observation		No of observations	Detected Outlier		Missing observation		No of observations
		ANC	SBA	ANC	SBA		ANC	SBA	ANC	SBA		ANC	SBA	ANC	SBA	
**North Central**	7	3 (1.1)	2 (0.8)	0 (0)	0 (0)	266	10 (8.4)	3 (2.5)	7 (5.9)	0 (0)	119	13 (3.4)	5 (1.3)	7 (1.8)	0 (0)	385
**North East**	6	6 (2.6)	7 (3.1)	0 (0)	0 (0)	228	12 (11.8)	1 (1.0)	12 (11.8)	0 (0)	102	18 (5.5)	8 (2.4)	12 (3.6)	0 (0)	330
**North West**	7	9 (3.4)	9 (3.4)	0 (0)	0 (0)	266	10 (8.4)	3 (2.5)	8 (6.7)	0 (0)	119	19 (4.9)	12 (3.1)	8 (2.1)	0 (0)	385
**South East**	5	6 (3.2)	5 (2.6)	0 (0)	0 (0)	190	7 (8.2)	1 (1.2)	5 (5.9)	0 (0)	85	13 (4.7)	6 (2.2)	5 (1.8)	0 (0)	275
**South South**	6	3 (1.3)	5 (2.2)	0 (0)	0 (0)	228	1 (1.0)	3 (2.9)	0 (0)	0 (0)	102	4 (1.2)	8 (2.4)	0 (0)	0 (0)	330
**South West**	6	2 (0.9)	3 (1.3)	0 (0)	0 (0)	228	9 (8.8)	0 (0)	7 (6.9)	0 (0)	102	11 (3.3)	3 (0.9)	7 (2.1)	0 (0)	330
Total	*37*	*29 (2.1)*	*31 (2.2)*	*0 (0)*	*0 (0)*	*1406*	*49 (7.8)*	*11 (1.7)*	*39 (6.2)*	*0 (0)*	*629*	*78 (3.8)*	*42 (2.1)*	*39 (1.9)*	*0 (0)*	*2035*
