# Supplementary figures and images for: Regional differences in the utilisation of antenatal care and skilled birth attendant services during the COVID-19 pandemic in Nigeria: an interrupted time series analysis
Source: BMJ Glob Health. 2023 Oct 21;8(10):e012464. doi: 10.1136/bmjgh-2023-012464 (PMC10603444; doi:10.1136/bmjgh-2023-012464)

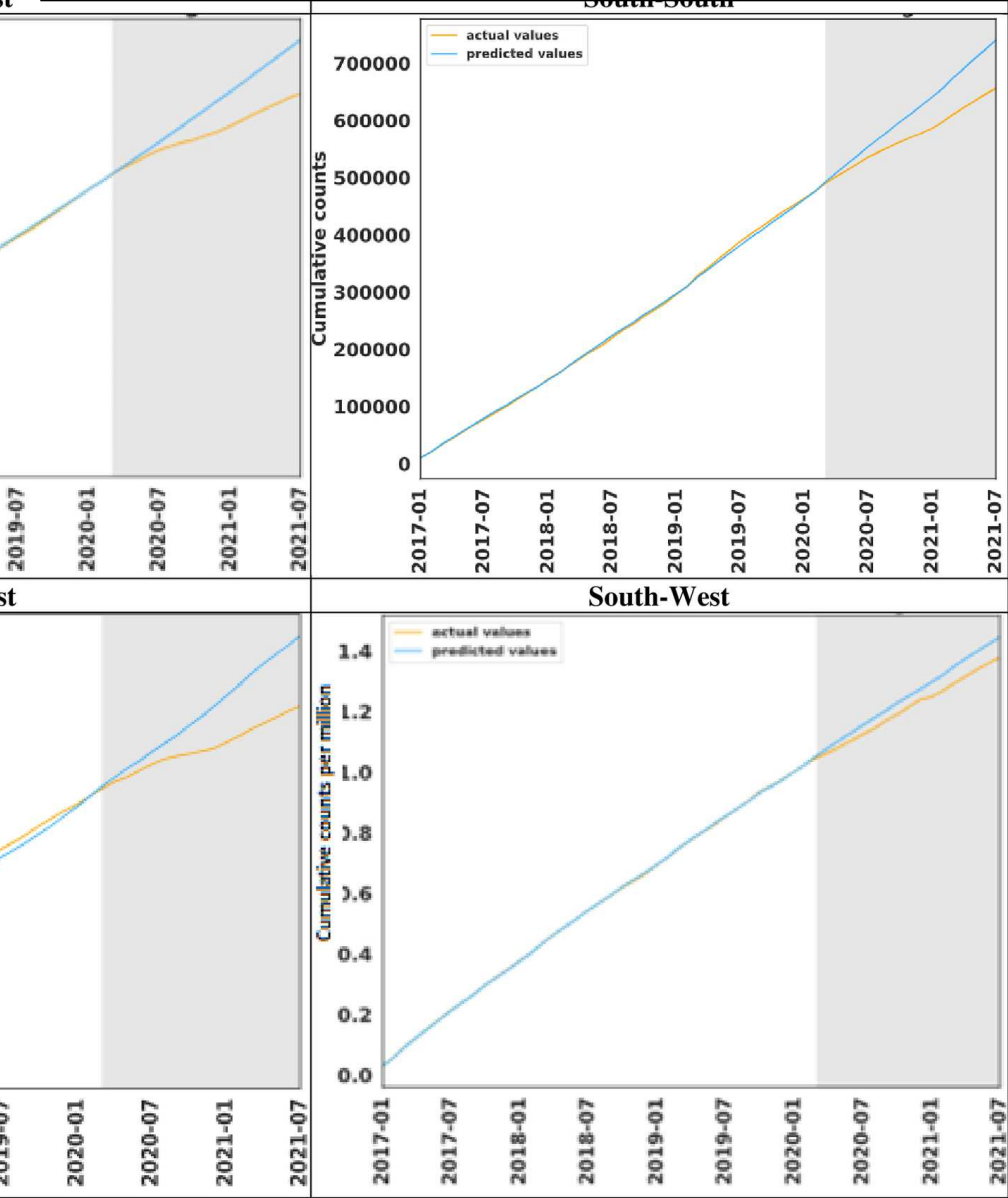

Supplement: Supplementary data [file bmjgh-2023-012464supp001.pdf]

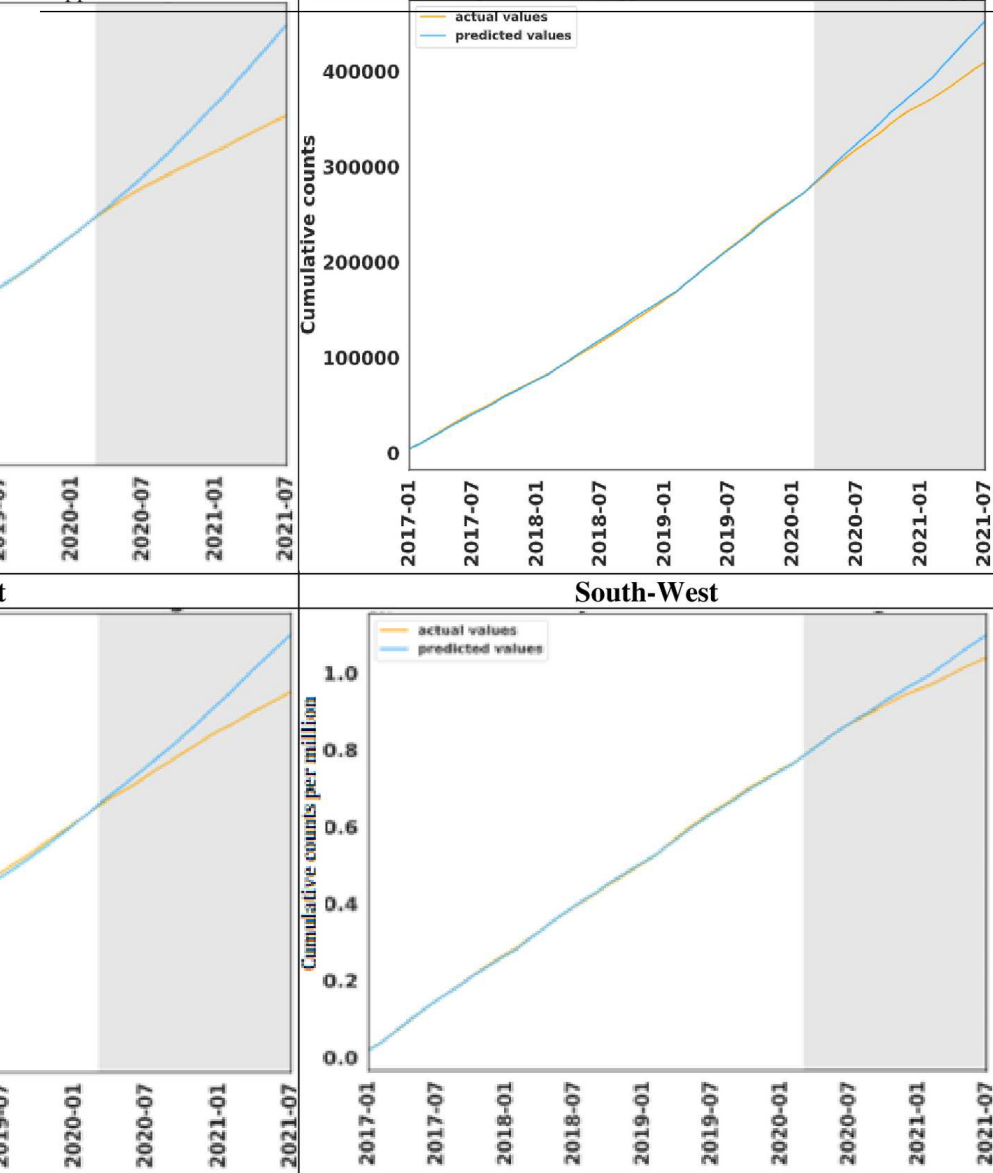

Supplement: Supplementary data [file bmjgh-2023-012464supp002.pdf]
